# Severe Central Hypoventilation Syndrome in a Patient With Anti-N-Methyl-D-Aspartate Receptor Encephalitis: Case Report and Review of the Literature

**DOI:** 10.7759/cureus.30749

**Published:** 2022-10-27

**Authors:** Maria Voronova, Artem Sharko

**Affiliations:** 1 Internal Medicine, I.M. Sechenov First Moscow State Medical University, Moscow, RUS; 2 Internal Medicine, Chicago Medical School at Rosalind Franklin University of Medicine and Science, North Chicago, USA; 3 Internal Medicine, Northwestern Medicine McHenry Hospital, McHenry, USA

**Keywords:** severe respiratory failure, autoimmune encephalitis, central hypoventilation syndrome, anti-nmda encephalitis, encephalitis

## Abstract

Anti-N-methyl-D-aspartate receptor (NMDAR) encephalitis is a condition that is characterized by a variety of psychiatric and neurological symptoms, including central hypoventilation syndrome (CHS). CHS presents with apnea or hypopnea and can vary in severity and rapidity of development but rarely leads to respiratory failure that requires mechanical ventilation. Our patient was diagnosed with anti-NMDAR encephalitis after he presented with classic symptoms, and cerebrospinal fluid analysis showed positive N-methyl-D-aspartate (NMDA) receptor antibodies. During the course of the disease, he developed CHS, which led to respiratory arrest. After treatment with corticosteroids, intravenous immunoglobulin, and plasma exchange, the patient’s symptoms improved with complete resolution of the apneic episodes.

## Introduction

Anti-N-methyl-D-aspartate receptor (NMDAR) encephalitis is an autoimmune encephalitis (AE) that usually presents with psychiatric or cognitive symptoms, seizures, speech changes, dyskinesia or abnormal tone, autonomic dysfunction, or a decrease in the level of consciousness. Diagnosis can be definitively made when at least one of the mentioned six major symptom groups is present and anti-NMDAR antibodies against the NR1 or NR2 subunit of the receptor are identified [1].

Central hypoventilation syndrome (CHS) is a condition that can present as apnea, hypopnea, and in severe cases, respiratory arrest due to poor coordination between the brainstem and respiratory muscles. CHS can occur due to different types of injury to the brain, including in anti-NMDAR encephalitis cases [2,3]. We present a rare case of severe and rapidly developing CHS in a 31-year-old male with anti-NMDAR encephalitis who required intubation.

## Case presentation

The patient is a 31-year-old male who presented to the emergency department (ED) due to altered mental status and visual and auditory hallucinations. He was with his wife, who witnessed him talking to people who were not present and expressing the desire to hurt people. The wife could not reorient him, so she brought him to the hospital.

Symptoms developed gradually over three months and initially included headaches, constant tremors in both hands, memory problems, and personality changes.

Two months before the current admission, the patient was brought to the ED for being unreasonably aggressive and severely agitated. He was evaluated with brain magnetic resonance imaging (MRI) and an electroencephalogram (EEG). The findings were unremarkable, so he was discharged.

He had a past medical history of diabetes mellitus type two and coronavirus disease 2019 (COVID-19) infection 14 months earlier. He had no surgeries in the past, and his family history was negative for any psychiatric or neurological conditions. The patient had no history of alcohol or recreational drug use. His only medication was metformin.

On physical examination, vital signs were within normal limits (WNL). He was only able to follow simple one-step commands intermittently. He appeared agitated and restless. Clonus and choreoathetoid movements were noted. His strength and reflexes were normal, and his tone was increased in all four extremities. Pupils were sluggishly reactive to light, but no other focal neurologic deficits were identified.

Initial complete blood count, complete metabolic panel, urine analysis, vitamin B12, folate, thyroid hormones, and cortisol were WNL. Urine drug screen and blood alcohol level were unremarkable. Erythrocyte sedimentation rate and C-reactive protein were elevated at 84 (reference range 0-15 mm/hour) and 6.3 (reference range <0.3 mg/L), respectively. The HIV test was negative. Blood cultures were pending. Computed tomography (CT) of the brain did not show any pathologic findings.

At this point, the differential diagnosis was broad and included infectious encephalitis, autoimmune encephalitis, multiple sclerosis, central nervous system vasculitis, and paraneoplastic syndrome.

A lumbar puncture was performed with analysis of cerebrospinal fluid (CSF) for cell count with differential, protein and glucose concentration, heavy metals, AE antibodies, oligoclonal bands, angiotensin-converting enzyme (ACE), stains and cultures, herpes simplex virus (HSV), arbovirus, cryptococcus, and syphilis. Furthermore, serum antinuclear antibodies (ANA), antineutrophil cytoplasmic antibodies (ANCA), cryoglobulin, double-stranded DNA antibodies, porphyrins, ceruloplasmin, and Lyme disease antibodies were sent.

To further investigate for possible sources of infection and potential tumors causing a paraneoplastic syndrome, a CT scan of the chest, abdomen, and pelvis and an ultrasound of the scrotum were performed and were unremarkable. Alpha-fetoprotein (AFP) and beta-human chorionic gonadotropin (HCG) were sent.

He was empirically started on broad-spectrum antimicrobials with vancomycin, cefepime, and acyclovir.

Within 48 hours, the patient’s vital signs became labile, with blood pressure (BP) fluctuating between 90/50 and 120/70 mmHg. Heart rate (HR) was 90 to 110 beats per minute, and the temperature was between 95.5 Fahrenheit (F) and 99.7 F. His saturation (SpO2) was 93-98% on room air, with a respiratory rate (RR) of 25 to 30 breaths per minute. An EEG was performed and demonstrated marked diffuse slow-wave abnormality compatible with an encephalopathic process but not specific for any etiology. The initial CSF analysis results became available (Table 1).

**Table 1:** CSF analysis results CSF - cerebrospinal fluid

Component	Lab value	Reference range
Total nucleated cells	107/μL	<5
Neutrophils	24%	0 - 6%
Lymphocytes	61%	40 - 80%
Monocytes	13%	14 - 45%
Other cells	2%	0%
CSF protein	73 mg/dL	15 – 45 mg/dL
CSF glucose	49 mg/dL	40 – 70 mg/dL

**Figure 1:**
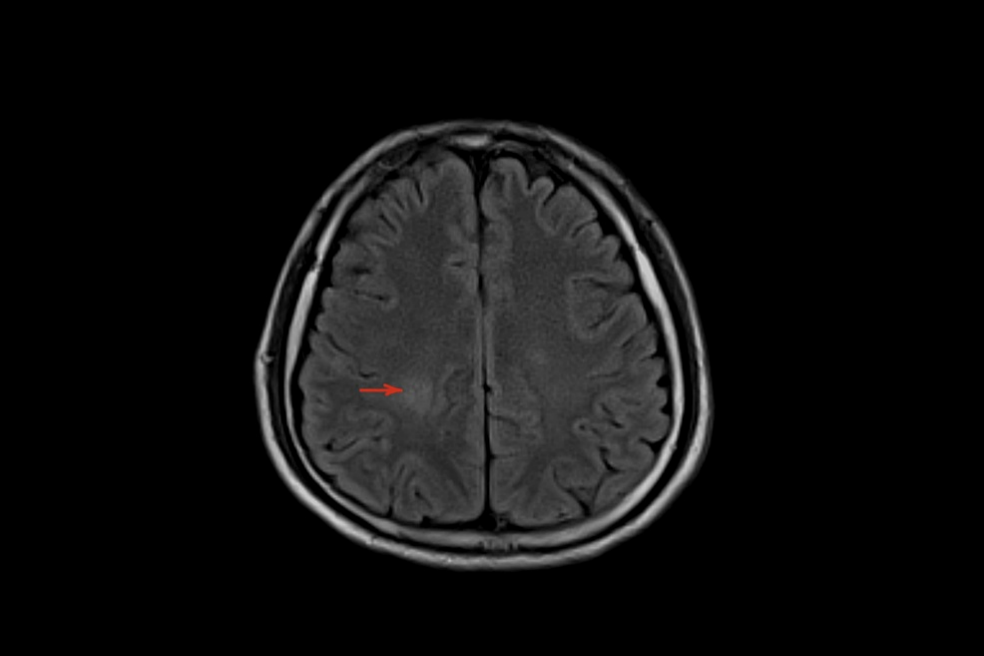
MRI of the brain: FLAIR signal hyperintensity in the right posterior parietal regions in the periventricular deep white matter (red arrow). MRI - magnetic resonance imaging; FLAIR - fluid-attenuated inversion recovery

On day three of hospitalization, he appeared less agitated but was hypothermic with a temperature of 95.5 F. An MRI of the brain showed a fluid-attenuated inversion recovery (FLAIR) signal hyperintensity in the parietal region (Figure 1), which was absent on the MRI performed two months prior.

On day five, hypothermia persisted with the temperature around 96.4 F. BP and HR remained labile. The RR was around 20-25 with normal SpO2. CSF cultures and blood cultures were negative. CSF workup for HSV, syphilis, arbovirus, and cryptococcus was also negative, so antibiotics and acyclovir were discontinued.

On day six, ANA resulted positive at 1:360. Based on the ANA and MRI findings, the suspicion for vasculitis or AE was high, so a five-day course of intravenous immunoglobulin (IVIG) for a total of 2 g/kg was initiated.

On day eight, the patient received his third dose of IVIG. He was still hallucinating but appeared calmer. Vital signs remained labile. Labs were negative for porphyrins, cryoglobulin, ceruloplasmin, CSF ACE, p-ANCA, and c-ANCA. Heavy metals, including arsenic, lead, mercury, cadmium, and mercury, were not detected. Vitamins B1 and B6 were WNL. AFP and beta-HCG were WNL. However, CSF oligoclonal bands and NMDAR antibodies against the NR1 subunit were positive.

On day nine, the patient started having frequent apneic episodes with severe hypoxia. During most episodes, the patient appeared to have been asleep and was snoring, but sometimes, he seemed to be awake with open eyes. Throughout these episodes, he would stop breathing, and his SpO2 would rapidly drop to as low as 30%. The episodes would continue until someone applied noxious stimuli, and after that, he would restart breathing, with SpO2 improving quickly to 95%. Initially, these episodes occurred every 20 to 25 minutes, but within two hours, the frequency gradually increased to every one to two minutes. He was placed on a bilevel positive airway pressure (BiPAP) machine, but the episodes persisted, and eventually, the frequency increased to approximately every 15 seconds, so he was intubated. A chest X-ray and a CT of the chest with the pulmonary embolism protocol were performed and did not show any pathologic findings. Due to the absence of pathologic processes in the lungs causing this worsening and the fact that the patient was already diagnosed with anti-NMDAR encephalitis, the most likely cause of these symptoms was CHS or severe obstructive sleep apnea (OSA). Obesity hypoventilation syndrome was excluded, as the patient's body mass index was 21. Because the patient had no history and no risk factors for OSA, and these symptoms occurred not just during sleep but also while awake, he was diagnosed with CHS. This acute worsening in the patient's condition was managed by continuing IVIG infusions and adding pulse dose steroids with methylprednisolone 1 gram (g) daily for five days.

Once IVIG and pulse dose steroids were completed, daily spontaneous breathing trials (SBT) were attempted. During SBT, he still had apneic episodes, although the frequency had decreased to approximately every 10 minutes. Due to insufficient response, on day 15, plasma exchange (PLEX) sessions every 48 hours were initiated.

On day 19, after three sessions of PLEX, agitation decreased, and he was able to follow basic commands. During SBT, he did not have any episodes of apnea; however, he remained intubated due to absent gag and cough reflex.

On day 23 of hospitalization, PLEX was completed. By this time, his mental status had improved significantly, he was following commands appropriately, and his gag and cough reflexes had improved. However, he had a generalized tonic-clonic seizure, which was managed with lorazepam, and he was started on prophylactic levetiracetam. EEG later that day showed severe generalized cerebral dysfunction and a delta brush pattern. No seizure activity was noted. Continuous EEG monitoring was started, and for the next three days, he remained seizure-free, continued to follow commands with gradual improvement of mental status, and his cough and gag reflexes normalized.

**Figure 2:**
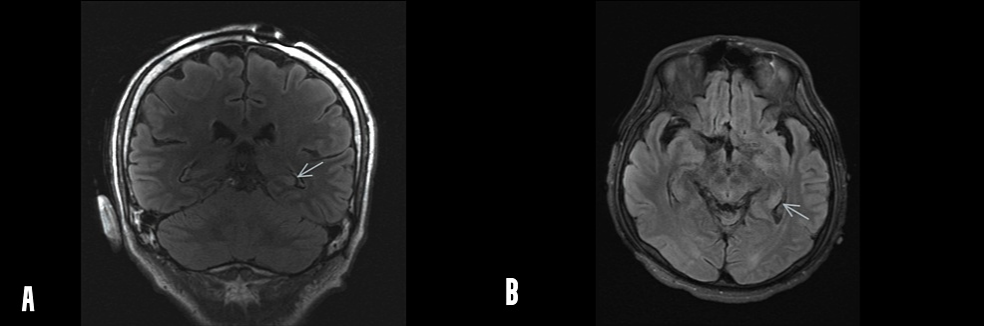
MRI of the brain: FLAIR signal hyperintensity within the dorsal body of the left hippocampus (blue arrow) in the coronal view (A) and axial view (B) MRI - magnetic resonance imaging; FLAIR - fluid-attenuated inversion recovery

On day 27 of hospitalization, the patient was extubated and transitioned to 3 liters of supplemental oxygen by nasal cannula with SpO2 ranging between 93% and 97%. No more periods of apnea occurred after extubation. A positron emission tomography (PET) scan was performed and did not show any evidence of occult malignancy. MRI of the brain was repeated and showed that new lesions in the hippocampus had appeared compared to the previous MRI (Figure 2).

The patient remained in the hospital for several more days and remained clinically stable. He was severely deconditioned and started working with physical and occupational therapy and eventually was discharged to an inpatient rehabilitation facility. Further management was planned to be continued outpatient under the care of a neuroimmunologist.

## Discussion

Anti-NMDAR encephalitis is a subtype of AE characterized by the presence of autoantibodies against NR1 or NR2 subunits of the N-methyl-D-aspartate (NMDA) receptor [4].

The disease can present in various ways, including seizures and movement disorders, such as chorea, athetosis, dystonia, myoclonus, tremor, or a combination of them. Autonomic disorders, such as vital sign lability, most commonly BP, HR, and T, can occur. Psychiatric symptoms, including hallucinations, agitation, and mood lability, are often present. Another common sign is cognitive impairment presenting as disorientation, confusion, and amnesia with confabulations. Cognitive symptoms are associated with lesions in the hippocampus [5]. Sleep disorders, such as insomnia, hypersomnia, and parasomnias, are a well-known presentation of patients with anti-NMDAR encephalitis and other AE. Parasomnias include sleep talking, sleepwalking, goal-directed behavior, and dream enactment [6]. Sleep apnea is also common in patients with AE [6]. A study that involved 26 patients with AE showed that 19 (73%) of patients developed new or worsening existent sleep disturbance after the diagnosis of AE. The most common sleep disturbances were snoring or gasping in nine (47%) and witnessed apneas in five (26%) cases. Among the nine patients with NMDAR encephalitis, five (56%) had sleep disturbances with new or worsening snoring, gasping, and sleep apneas seen in two (22%) of them [6]. After treatment with immunosuppressive medications, five out of nine (56%) patients with AE reported improvement or resolution of snoring, gasping, and apneas [6].

Often, psychiatric symptoms are the initial complaint motivating patients to seek psychiatric help, which frequently guides clinicians to the wrong diagnosis. Movement disorders, seizures, and other symptoms usually appear later in the course of the disease, which prompts further investigation that leads to the final diagnosis [5].

CHS is a condition characterized by abnormal breathing patterns, such as apnea, hypopnea, and in severe cases, respiratory arrest. It occurs due to autonomic dysfunction leading to ineffective coordination between the respiratory center in the brainstem and the respiratory muscles [2]. CHS is usually congenital and associated with paired-like homeobox 2B (PHOX2B) gene mutations [7]. However, it can also occur due to different types of injury to the brain such as brainstem infarctions, central nervous system infections, brain tumors, and autoimmune diseases [2]. CHS presents as a decrease in respiratory rate and tidal volume leading to hypoventilation, which is characterized by hypoxemia and hypercapnia that does not result in an appropriate response from the respiratory center [7]. The clinical symptoms are more apparent during sleep, especially during non-rapid eye movement (NREM) sleep. Diagnosis is initially clinical and can be definitively made by polysomnography and cardiorespiratory polygraphy, which show episodes of hypoventilation that are more frequent during NREM sleep than rapid eye movement (REM) sleep and wakefulness [7]. A retrospective study showed that CHS occurs in up to 25% of anti-NMDAR encephalitis cases [3]. However, it is predominantly very mild and associated with only a slight drop in SpO2. Cases that require mechanical ventilation are extremely rare.

Anti-NMDAR encephalitis can present as a paraneoplastic syndrome and is known to be associated with ovarian teratomas. An observational study of 577 anti-NMDAR encephalitis patients showed that 220 (38%) of them had a neoplasm. A neoplasm was identified in 46% of females and only 6.1% of males. Of all tumors identified in females, 97% were ovarian teratomas [8].

First-line therapy includes pulse dose corticosteroids with methylprednisolone 1000 mg daily for three to five days, IVIG 2 g/kg administered over five days, and PLEX sessions every 48 hours for a total of five to seven cycles. Tumor removal, when one is identified, is essential in the management of the disease [9].

Second-line therapy includes rituximab 375 mg/m^2^ weekly infusion for four weeks or cyclophosphamide 750 mg/m^2^ monthly for three to six months [9].

Usually, the initial choice is corticosteroids with or without IVIG. Studies have shown that PLEX given after steroids leads to better outcomes when compared to steroids alone [9].

When first-line therapy does not lead to the desired outcome, second-line agents are used. A study divided 161 patients who completed first-line therapy into two groups. The treatment group received rituximab, and the control group did not. The group that received rituximab failed to show a better modified Rankin Scale (mRS) score than the control group during the last follow-up and did not achieve the mRS goal, which is considered to be between zero and two. However, after that, the patients were divided into two groups based on their response to first-line therapy [10]. Among the 82 patients who had no response at all to first-line therapy, 55 received rituximab, and 33 (60%) of them achieved the mRS goal of zero to two, while among the 27 that did not receive rituximab only six (22,2%) achieved the mRS score goal [10]. This result indicates that rituximab may benefit patients who had absolutely no response to first-line therapy. Cyclophosphamide is another second-line agent; however, its use is limited due to severe side effects [9].

An observational study that included 501 patients showed that among the 472 who were treated with first-line therapy and tumor removal, 251 (53%) achieved clinical improvement, and 241 of them reached the mRS score goal within 24 months. No improvement with first-line therapy was noted in 221 (47%) patients; among them, 125 received second-line therapy, with 84 out of 125 (67%) reaching the goal of the mRS score within 24 months. Ninety-six of the 221 patients did not receive second-line therapy, and 49 (51%) of them achieved the goal of the mRS score within the same period of time [8]. 

If first or second-line therapy does not lead to desired outcomes or the patient cannot tolerate the medications, alternative options can be utilized. Alternative options include tocilizumab, usually started at 4 mg/kg, with a subsequent increase to 8 mg/kg monthly [9]. A retrospective study showed that tocilizumab administered to patients who failed first-line therapy and rituximab led to desired outcomes of the mRS score of zero to two more frequently than a maintenance dose of rituximab or no treatment [11]. An alternative option is low-dose interleukin-2 therapy, which showed improvement of mRS in patients with AE refractory to first and second-line therapy, especially anti-NMDAR encephalitis [12]. Bortezomib has also been discussed as a potential treatment option for anti-NMDAR encephalitis, and reports have shown that it can lead to clinical improvement, however, more studies are needed to make conclusions about its effectiveness [9].

Anti-NMDAR encephalitis is characterized by relapses in approximately 12% of cases. Many patients have more than one relapse [8]. In order to prevent relapses, maintenance therapy is often utilized. Long-term monthly doses of rituximab can prevent relapses and further improve the mRS score [10]. Maintenance dose of oral corticosteroids, intermittent IVIG infusion, or PLEX has also been used [9]. If steroids are contraindicated or not tolerated, azathioprine or mycophenolate mofetil can be used as alternative options [9].

Multiple factors determine the prognosis of anti-NMDAR encephalitis. The most predictive ones include the need for intensive care unit (ICU) admission, promptness of appropriate treatment initiation, and tumor removal when one is present [8].

Diagnostic evaluation of patients presenting with symptoms suspicious for anti-NMDAR encephalitis includes neurological imaging, EEG, and CSF analysis. Definitive diagnosis can only be established in the presence of anti-NMDAR antibodies against NR1 or NR2 subunits of the NMDA receptor and at least one of the six major symptom groups that include psychiatric or cognitive symptoms, seizures, speech changes, dyskinesia or abnormal tone, autonomic dysfunction or central hypoventilation, and decrease of the level of consciousness [1].

Imaging and EEG help differentiate between psychiatric and organic causes of the symptoms. Certain findings can increase suspicion for anti-NMDAR encephalitis and help with prognostication of the clinical course. An EEG finding specific to anti-NMDAR encephalitis is the “extreme delta brush” (EDB) pattern. EDB pattern is frontally predominant delta activity, which is symmetric and synchronous with overriding fast activity [1,13]. Severe generalized slowing is another typical pattern seen in these patients, characterized by delta range frequencies [13,14]. Both EDB and severe generalized slowing are associated with a worse prognosis and a higher likelihood of admission to the ICU. Seizure activity is a rare finding on EEG in anti-NMDAR encephalitis, even though seizures are common [13].

Regarding imaging, MRI of the brain is the test of choice. Findings are usually nonspecific but can assist in excluding many differential diagnoses. In patients with anti-NMDAR encephalitis, brain MRI usually shows hyperintensities on the FLAIR sequence located at the periventricular region, temporal, and frontal lobe [14,15]. A study conducted to determine the most common MRI findings in anti-NMDAR encephalitis showed that 53% of patients had normal MRI. Of the patients with abnormal MRI, 28% had lesions located only in the hippocampus, and 28% had lesions in areas other than the hippocampus. The most common areas are the frontal or temporal lobe, cingulate gyrus, middle cerebellar peduncle, corpus callosum, insula, basal ganglia, thalamus, or brain stem. The remaining 44% had lesions both in the hippocampus and in other areas of the brain. The study showed that hippocampal lesions are associated with poor prognosis and worse outcomes [16].

## Conclusions

Anti-NMDAR encephalitis is a rare medical condition that has been studied and thoroughly described. On the other hand, CHS is not a commonly encountered presentation of the disease, and it has not been described well enough to guide clinicians in its management. In our case, the rapidity of the development of the syndrome and its severity were unique. CHS, in its most severe form, can be life-threatening, and if not recognized and managed in a timely manner, it can lead to fatal outcomes. We believe that physicians should be aware of this condition and be able to recognize its signs. Every patient with diagnosed or suspected anti-NMDAR encephalitis should be placed on continuous pulse oximetry and end-tidal carbon dioxide (CO2) monitoring. If signs of hypoventilation and respiratory failure become obvious, we recommend that such patients be transferred to the ICU immediately for closer monitoring of the respiratory status and intubation if deemed necessary.
